# Longitudinal outcomes of different asthma phenotypes in primary care, an observational study

**DOI:** 10.1038/s41533-017-0057-3

**Published:** 2017-10-03

**Authors:** Rishi J. Khusial, Jacob K. Sont, Rik J. B. Loijmans, Jiska B. Snoeck-Stroband, Pim J. J. Assendelft, Tjard R. J. Schermer, Persijn J. Honkoop, for the ACCURATE Study Group

**Affiliations:** 1 Dept of Medical Decision Making, Leiden University Medical Center, Leiden, The Netherlands; 2 Dept of General Practice, Academic Medical Center, Amsterdam, The Netherlands; 3 Dept of Primary and Community Care, Radboud University Medical Center, Nijmegen, The Netherlands

## Abstract

While asthma presentation is heterogeneous, current asthma management guidelines in primary care are quite homogeneous. In this study we aim to cluster patients together into different phenotypes, that may aid the general practitioner in individualised asthma management. We analysed data from the ACCURATE trial, containing 611 adult asthmatics, 18–50 year-old, treated in primary care, with one year follow-up. Variables obtained at baseline (*n* = 14), were assessed by cluster analysis. Subsequently, established phenotypes were assessed separately on important asthma outcomes after one year follow-up: asthma control (Asthma Control Questionnaire (ACQ)), quality of life (Asthma Quality of Life Questionnaire (AQLQ)), exacerbation-rate and medication-usage. Five distinct phenotypes were identified. The first phenotype was predominantly defined by their early onset atopic form of asthma. The second phenotype mainly consisted of female patients with a late onset asthma. The third phenotype were patients with high reversibility rates after bronchodilator usage. The fourth phenotype were smokers and the final phenotype were frequent exacerbators. The exacerbators phenotype had the worst outcomes for asthma control and quality of life and experienced the highest exacerbation-rate, despite using the most medication. The early onset phenotype patients were relatively well controlled and their medication dosage was low.

## Asthma: Individualizing treatment by phenotype

Asthma patients should be characterised according to their individual asthma type to ensure more targeted treatment. Even though asthma manifests itself in a wide variety of forms with differing degrees of severity, treatment of the disease often takes a broad, one-size-fits-all approach. To determine if asthma can indeed be split into distinct phenotypes, Rishi Khusial at the Leiden University Medical Center and co-workers across the Netherlands analysed data from 611 adult asthmatics treated in primary care, and followed them up after one year. The team identified five phenotypes in the primary care cohort, including one group with early onset asthma, another whose asthma responded well to bronchodilators, and a group classed as frequent exacerbators. Further analysis of long-term asthma outcomes showed clear differences between phenotypes, particularly in terms of asthma control and quality of life.

## Introduction

Over 300 million people suffer from asthma globally, which makes it one of the most common chronic diseases in the world.^1^ International guidelines such as the Global Initiative for Asthma formulate the long-term goals of asthma treatment as the accomplishment of asthma control and reducing the exacerbation rate.^2,3^ Although people with asthma generally perceive their asthma control positively, the level of actual clinical asthma control in Europe fails to reach these goals.^4–7^ Part of the reason these goals are not met, is that asthma is a heterogeneous disease, while management and treatment according to current guidelines is, despite its step-wise approach, more of a one-size-fits-all approach, that is not personalised according to individual patient characteristics. Tailored interventions using FeNO^8^ or sputum eosinophils^9^, for example have proven successful in reducing steroid dose and exacerbation rate, respectively.

Currently, several different asthma phenotypes that cluster specific patient characteristics have been described.^10–13^ Managing asthma patients according to these phenotypes might be an approach that allows for individualising treatment. Thus far most of the studies identifying phenotypes have been performed in hospital care. However, in many countries, the majority of the individuals with asthma are treated in a primary care setting.^14–16^ Therefore, it is important to assess whether these phenotypes are similar in primary care. Additionally, the majority of the current hospital care-based phenotypes require certain measurements not easily available in primary care. This hampers implementation and it would be preferable if primary care phenotypes consist of measurements easily obtainable to the general practitioner (GP) or practice nurse (PN). Therefore, as the first part of our study we aimed to cluster primary care asthma patients into distinct phenotypes based on easily obtainable patient characteristics using a clustering strategy.

Furthermore, continuing onward with these phenotypes, efficiency of asthma management might be improved if it is known which phenotypes require more frequent assessments by a GP or PN, because they present a greater risk of future adverse outcomes and which phenotypes could be safely assessed less frequently due to more favourable long-term asthma outcomes. Therefore, in the second part of our study we determined the long-term differences in important asthma outcomes between different phenotypes. We aimed to assess differences in our established phenotypes in terms of asthma control, quality of life, medication usage and exacerbation frequency, after a 12-month follow-up period.

## Results

The results described here were generated in a five step process, which is described in detail in our methods section. In the first step we assessed potential variables to be included in our cluster analysis and these are listed in Table 1. Overall, our population is comparable to the general asthma population in primary care in Europe, including a higher percentage of females (Table 1).^3,17^

**Table 1:** Baseline data of total research population

Variable	Total (*n* = 611)
Gender (% female)^a^	68.4
Age, yr (SD)^a^	39.4 (9.1)
Age of diagnose, yr (SD)^a^	20.8 (14.4)
Length, cm (SD)	172.4 (10.1)
Weight, kg (SD)	79.0 (17.7)
BMI, kg/m^2^ (SD)^a^	26.4 (5.3)
ACQ6^a^	1.0
AQLQ	5.7
Reversibility, %^a,b^	6.6
Active smokers (% patients)	14
Pack years smoked, yr^a^	4.7
Exacerbations in past 12 months, no per patient^a^	0.67
FeNO^a^	23.3
FEV1 (% predicted)^a^	95

^a^ Variable used in cluster analysis

^b^ Percentage reversibility accomplished after use of a bronchodilator

Next, we performed a factor analysis which showed four distinct factors. These factors were defined by: (1) the level of self-reported patient health, labelled ‘functioning’; (2) the combination of prescribed medication, labelled ‘medication’; (3) the atopic status, labelled ‘allergy’ and (4) the socioeconomic status, labelled ‘SES’. The results of the factor analysis are reported in Table 2, results are standardised *z*-scores and positive scores represent an above average result (see methods). Subsequently in step 3 data transformation was performed for selected variables. FEV1 was adjusted for gender and missing variables were imputed. Then we performed a cluster analysis with 14 variables (including factors), all variables were continuous except gender.

**Table 2:** Clustering results

Variable	Cluster 1	Cluster 2	Cluster 3	Cluster 4	Cluster 5
	(*n* = 170)	(*n* = 236)	(*n* = 62)	(*n* = 62)	(*n* = 81)
	Early atopic	Late onset female	Reversible	Smokers	Exacerbators
Gender (%female)	45.3	85.2	75.8	51.6	75.3
Age, yr	34.8	42.5	34.5	44.5	39.7
Age of diagnose, yr	13.7	27.1	16.5	23.5	18.5
BMI, kg/m^2^	25.2	27.4	25.1	25.5	27.5
ACQ6	0.5	0.9	1.7	1.0	1.6
AQLQ	6.3	5.6	5.3	5.8	5.1
Reversibility, %	−1.8	2.7	35.1	10.7	10.9
Pack years smoked, yr	1.2	2.9	2.1	24.0	4.4
Exacerbations in past 12 months, no per patient	0.1	0.4	0.5	0.4	3.0
FEV1 (% predicted)	96	100	70	89	99
Factors^a^					
SES	0.20	0.01	−0.16	−0.48	0.03
Allergy	0.27	−0.20	0.05	−0.33	0.22
Functioning	0.42	−0.18	−0.16	0.15	−0.37
Medication	−0.38	0.18	0.05	0.01	0.24

^a^ Factor scores are standardised *Z*-scores with mean = 0 and standard deviation = 1

An agglomerative hierarchical cluster analysis with baseline data showed that the optimal amount of clusters was five. A general overview of the clusters is presented in Table 2. In summary, the first phenotype consisted of patients with an early diagnosis of asthma and a high prevalence of allergies. This phenotype also reported the highest functioning scores and socio–economic status (SES). Most patients in this phenotype were male. For further reference, this phenotype was labelled ‘early atopic’.

The second phenotype, labelled ‘late onset female’, was the largest group. It consisted mainly of females and the mean age of diagnosis was slightly higher than in the other phenotypes.

The third phenotype, labelled ‘reversible’, consisted of patients with high reversibility after bronchodilation and the lowest baseline FEV1 percentage predicted. This group also reported the most symptoms in the Asthma Control Questionnaire (ACQ6).

The fourth phenotype, labelled ‘smokers’, included patients with the highest amount of pack years smoked. This not necessarily meant the patients were still active smokers during the study (a total of 48% was actively smoking). This group also reported the lowest SES.

The fifth and last phenotype, labelled ‘exacerbators’, comprised of patients who frequently experienced exacerbations in the year previous to the study. They also reported relatively high ACQ scores.

### Long-term outcomes

Finally we analysed long-term outcomes of important asthma parameters for each of the phenotypes.

#### ACQ

**Fig. 1:**
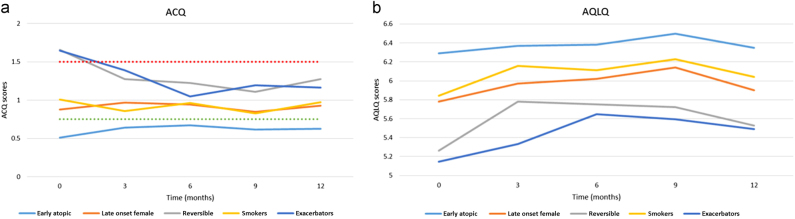
**a**. ACQ score in a 12-month study period divided per cluster. Time period 0 is the baseline measurement. A high ACQ scores stands for less controlled asthma. ACQ-scores can be divided in: controlled (ACQ score ≤ 0.75, below green dotted line), partly controlled (0.75 < ACQ score < 1.5), or uncontrolled (ACQ score ≥ 1.5, above red dotted line). The clusters started out with different baseline scores. The ‘exacerbators’ and ‘reversible’ phenotypes started as uncontrolled, but after 12 months progressed to partly controlled. The ‘smokers’ and ‘late onset female’ managed as partly controlled.^33^ The only group with controlled asthma is the early atopic group. **b**. AQLQ scores in a 12-month study period divided per cluster. Time period 0 is the baseline measurement. Higher AQLQ scores mean a better quality of life. The ‘late onset female’ and the ‘smokers’ have comparable starting and ending values, and a small progress can be noted for both groups. The reversible and exacerbation phenotypes have the highest increase in quality of life in the 12-month study period, albeit still lower than the other phenotypes. In all groups a decrease in scores can be noted if month 12 is compared to month 9

Figure 1a presents the results of the ACQ during 12-month follow-up for each of the phenotypes. Data at baseline (month 0) were used in the cluster analysis to determine the phenotypes. The ‘reversible’ and the ‘exacerbators’ phenotypes had the most symptoms at baseline, showed the most improvement during follow-up, but at 12-month follow-up still reported most symptoms. The ACQ score of the other clusters were relatively stable over time and fluctuated around the results of the baseline measurement. The ‘late onset female’ and the ‘smoker’ phenotypes had very similar overall results. The ‘early atopic’ phenotype experienced a clinically meaningful lesser amount of symptoms throughout the study (defined as exceeding the minimal important difference (MID) of 0.5), compared to the ‘reversible’ and ‘exacerbators’ phenotypes (ACQ-results respectively 0.63, compared to 1.26 and 1.17).^18^

#### Asthma quality of life questionnaire (AQLQ)

Over the 12-month follow-up, all phenotypes gradually improve in quality of life over time, with the ‘exacerbators’ phenotype showing the most improvement. After 12-month follow-up the ‘early atopic’ phenotype has the best asthma related quality of life (AQLQ = 6.35). This was >0.5 points above both the ‘reversible’ (5.53) and the ‘exacerbators’ (5.49) phenotype, again indicating a clinically meaningful MID for the AQLQ.^19^ The AQLQ-results of the different phenotypes never cross each other.

#### Exacerbations

**Fig. 2:**
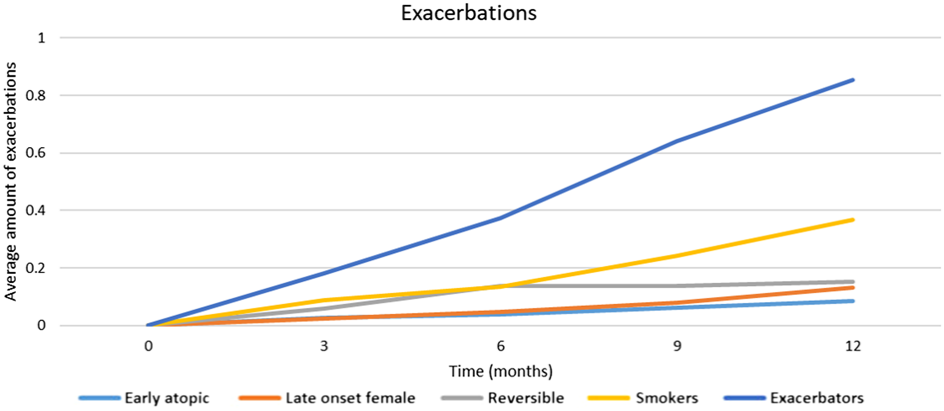
Cumulative amount of exacerbations in a 12-month study period divided by cluster. At 3-month interval, the total amount of exacerbations experienced were added in a cumulative score for every phenotype. The trend of the exacerbation progression over time is close to linear

In Fig. 2 the cumulative mean amount of exacerbations during the study is presented. The ‘exacerbators’ phenotype experienced the highest absolute amount of exacerbations in the follow-up period. The ‘smoker’ phenotype also had a relative high amount of exacerbations compared to the other phenotypes.

#### Medication usage

**Fig. 3:**
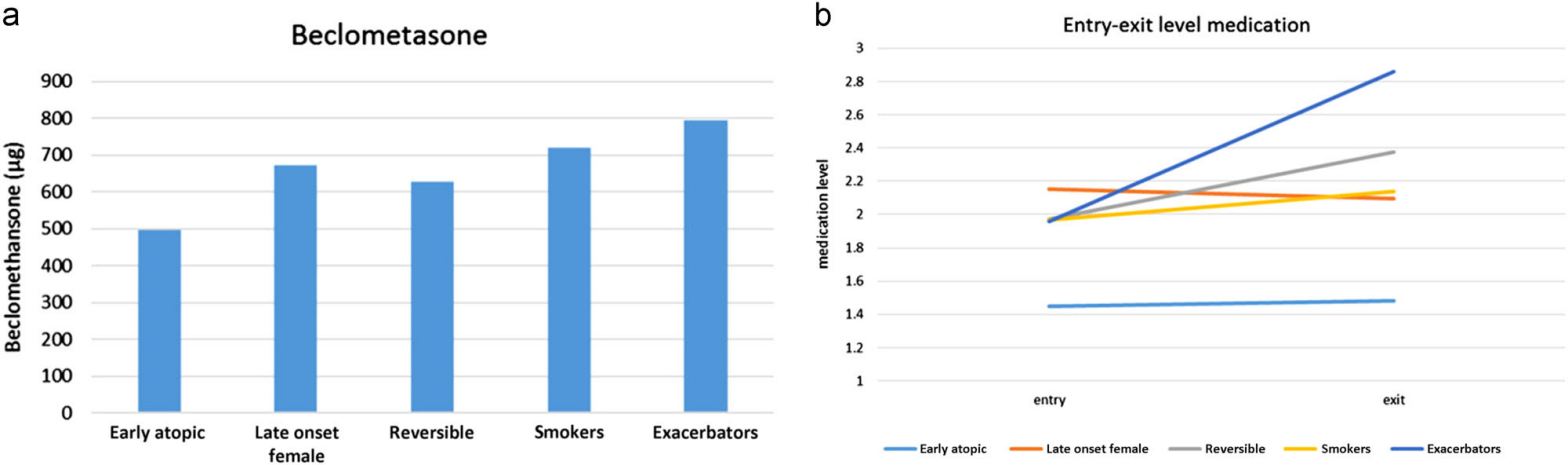
**a** Medication dosage at 12 months. All inhalation corticosteroid medication usage has been converted to beclomethasone equivalent. The ‘exacerbators’ phenotype had the highest dosage beclomethasone (794 µg) and the ‘early atopic’ group had the lowest dose (496 µg). The reversible group had an average dose of 628 µg. **b** Medication entry–exit level. This figure shows the mean guideline derived medication usage for each phenotype, with steps from 0 (no medication) to 5 (daily oral prednisolone).^26^ Over time it can be noted that while most phenotypes stayed roughly in the same medication step, the mean increase in medication usage for the ‘exacerbators’ phenotype was nearly one step. Phenotype ‘reversible’ also had a considerate increase in medication usage (from 2.0 to 2.4)

Figure 3a shows the total dosage of inhaled corticosteroid (ICS) after 12-month follow-up for each of the phenotypes and Fig. 3b shows the change during the study in guideline derived treatment steps.^2^ The ‘exacerbators’ phenotype were prescribed the highest ICS dosage after 12 months and also increased their dosage the most during the study. The ‘early atopic’ phenotype were prescribed the lowest dosage from start to finish.

## Discussion

### Main findings

In this study we established five distinct asthma phenotypes in primary care. Important long-term asthma outcomes, such as asthma control, quality of life, exacerbation rate and medication usage differed between these phenotypes after 12-month follow-up. Therefore, taking into account asthma phenotypes allows for a more personalised treatment approach and in so doing can have important consequences for long-term asthma outcomes.

### Interpretation of findings in relation to previously published work

In our study in primary care we established five distinct asthma phenotypes. Previously, Haldar et al.^11^ found three clusters in a primary care population, namely: ‘early-onset atopic asthma’, ‘obese noneosinophilic’ and ‘benign asthma’. In our study the ‘early-onset atopic’ phenotype had the most favourable outcomes, in terms of asthma control, quality of life and exacerbations, while using the lowest dosage of medication. This is in contrast to previous studies that suggest that early onset atopic patients use higher ICS dosage than other phenotypes.^20^ This can be attributed to our additional specific ‘exacerbators’ phenotype, which can be seen as the subset of patients with the worst long-term asthma outcomes. Therefore, by using our phenotypes, patients with the worst long-term outcomes are separated from the other phenotypes and consequently treatment can be more targeted to those with an increased risk.

The clusters in the study by Ortega^21^ showed, despite the high average body mass index (BMI), some familiarities with our clusters. Cluster 3 has relative many allergic patients but since this is not the group with the youngest patients, this does not directly corresponds with our ‘early onset atopic’ phenotype. Cluster 7 could correspond with our ‘exacerbators’ phenotype and cluster 5 could correspond with our ‘smokers’ phenotype. However, acknowledging the differences in variables used, the clusters are not directly the same.

An important reason that we found several different phenotypes from previous studies was that we added variables easily assessed in primary care. We also included FeNO as a variable in our analyses as it is now available in primary care and is shown to be cost-effective.^22^ We also included the variable pack years smoked in the cluster analysis. The amount of pack years smoked highly influences respiratory disease progression. This is somewhat reflected in the ‘smokers’ phenotype with a higher exacerbation rate. In contrast to previous studies, we did not detect one specific cluster of obese patients.^11,20^ One possible explanation could be the relatively high mean BMI (26.4) in our population, which allows for less distinction purely based on weight. This high mean BMI is comparable with the average BMI in western society.^23–25^

### Strengths and limitations

An important strength of our study is that we specifically studied an asthma population managed in primary care, while most former studies assessed phenotypes in hospital care. In our study we included many patients (*n* = 611) and we had 12-month follow-up data. We specifically used variables that were easily measurable and did not look at eosinophilic airway inflammation and comparable laboratory-requiring variables.

Another strength of our study is the heterogeneous group of primary care patients, of which many different variables were collected during the original trial, which allowed us to use many variables for clustering. The variable selection and analyses were based on results from previous literature.

A limitation of our study was that the differences within a phenotype (from baseline to 12 months) were usually higher than differences between phenotypes (at a certain point). While the ‘exacerbators’ phenotype had the relative highest risk of developing an exacerbation in the future, the absolute amount of exacerbations they experienced declined in the study year (mean 0.85 exacerbations per patient per year (exac/pt/yr)), compared to the year prior to the study (3.0 exac/pt/yr). This can partly be attributed to the beneficial effect of participating in a clinical trial, since the total amount of exacerbations in the whole research population also decreased (0.67 to 0.24 exac/pt/yr).

Another limitation of our study was the amount of patients per phenotype. Even though our general asthma population comprised of many patients, some phenotypes were too small (smallest phenotype *n* = 62) to perform further analysis, which resulted in our study having a more descriptive character. Further studies require more patients per phenotype to perform optimal treatment strategy analyses.

In our study we used the data set from the ACCURATE trial, in which patients were randomised into three different treatment strategies (‘partially controlled’, ‘controlled’ and ‘FeNO’).^22,26^ Our phenotypes were determined using baseline data from this trial, which were obtained before randomisation. Therefore the treatment strategies of the original trial had no influence on the establishment of the phenotypes. However, long-term outcomes could have been influenced if one of the treatment strategies of the original trial would have been overrepresented in one of our phenotypes. Therefore we performed a sub-analysis and this showed that treatment strategies were roughly equally distributed amongst the phenotypes (data not shown).

### Implications

Determining different phenotypes is of aid in identifying patients at risk, enabling primary care physicians to guide management, including frequency of control visits, treatment decisions and proper allocation of patients to primary care or hospital care management. To date, to describe people with asthma, usually mean scores are used, while the spread of scores is high. This makes it hard to make general statements about asthma patients in primary care and agglomerating all these different patients together leads to ineffective use of the healthcare system. Differentiating by using phenotypes might tackle this problem and the differences in long-term outcomes between the phenotypes in our study show that they function differently from one another, which underscores the benefit of phenotyping asthma patients in primary care.

For example, the ‘exacerbators’ phenotype clearly has the least favourable outcomes, even though they are prescribed the highest level of medication. Therefore, only increasing medication dosage for these patients is insufficient. Even though exacerbation rate improved in comparison to the year before the study, it is still high, while patients might experience additional side-effects. This suggests these patients additionally require a more thorough review of other (non-pharmacological) treatment options and if no clear improvements can be made, they should probably be referred to specialised care.^26^ On the other side of the spectrum is the early onset atopic phenotype. This group had good results on both ACQ and AQLQ and they had the lowest medication level. Patients with this phenotype show stable and favourable long-term outcomes and they are potentially suited to a more ‘as necessary’ approach to asthma management, for example by using online self-management through eHealth, alongside ‘as necessary’ contact with a healthcare provider.^27^ Similarly, the 'late onset female' phenotype, also shows more favourable outcomes. Together these two phenotypes comprise 66% of the total study population. It would be beneficial for both patients and the healthcare system as a whole if visits to the GP-office for these phenotypes could be limited.

Further research should focus on the implementation of phenotype usage in primary care. Firstly it must be assessed how to easily define a particular patients’ phenotype within current asthma management and how often this should be repeated in order to continually improve long-term asthma outcomes.^13^ Also, while we did show long-term outcomes are different for phenotypes, it is currently not certain that changing therapy according to this knowledge will actually lead to improved outcomes. Although this seems likely, a future prospective study is needed, which could also assess the consequences of implementing phenotypes in general practices, since if it is time-consuming while real life impact on long-term outcomes are negligible, use of phenotypes is questionable.

## Conclusions

Clustering of primary care asthma patients resulted in five distinct phenotypes. These phenotypes differ in long-term outcomes, as measured by ACQ, AQLQ, exacerbation frequency and medication usage. Using these phenotypes in asthma treatment may result in a personalisation of treatment, based on individual long-term outcomes.

## Methods

### Subjects

For this study we used data collected in the Asthma Control Cost-Utility Randomised Trial (ACCURATE), a multicentre randomised trial performed in the Netherlands. The goal of this original study was to assess three different treatment strategies of asthma.^22,28^ The results of this trial have previously been published. For a detailed description of study procedures and participants see the published protocol.

In short, participants were selected in primary care offices in both rural and urban areas in the Netherlands. All patients gave informed consent. Inclusion criteria were a doctors diagnosis of asthma, age 18–50 year-old, and a prescription for inhalation corticosteroid’s (ICS’s) for at least 3 months in the previous year. Patients were excluded if they had used an oral corticosteroid in the month prior to the study, were unable to speak Dutch or suffered from severe comorbidity. Methods were performed in accordance with relevant regulations/guidelines. The trial was approved by the Medical Ethics Committee of Leiden University Medical Center and registered at http://www.trialregister.nl/trialreg/admin/rctview.asp?TC=1756.

### Study design

The ACCURATE study was a cluster randomised trial with a follow-up of 12 months. For the establishment of the phenotypes baseline data were used, which contained data of patients before randomisation altered outcomes. Participants were reviewed every 3 months and if necessary treatment was adjusted. Additionally patients filled out online questionnaires. For our study we retrospectively analysed the data. Long-term outcomes of phenotypes were assessed using the data from the three monthly assessments and online questionnaires.

This analysis in this study consisted of the following five steps:

1. Identification of potentially relevant variables
2. Factor analysis
3. Data transformation, if necessary
4. Cluster analysis
5. Comparison of long-term outcomes

In the first step all potentially relevant variables were identified based on previous studies and relevant guidelines.^2,11,29^ General patient characteristics (gender, age, BMI, income and education level) and asthma specific variables were selected. All variables used for the analysis are listed in Table 1. To determine the different phenotypes, baseline data were used to approximate real life circumstances.

In the second step, we performed a factor analysis. A factor analysis was conducted before clustering to combine interrelated variables that measure the same construct, namely: SES, allergy, functioning scores on the sf36 questionnaire and medication. A varimax rotation was used to maximise variance.^30^

In the third step, part of the data was transformed to increase validity of the analysis and FEV1 was adjusted for gender and extreme outliers. Data were imputed if there were missing values at random, with STATA-command imputation. Additionally all variables used for clustering were standardised, with a mean of 0 and a standard deviation of ±1 in order to give all variables equal weight in the cluster analysis.

The fourth step consisted of a Wards linkage cluster analysis.^31^ This is the most optimal agglomerative hierarchical clustering strategy for the given data. Following Ward’s criterion for minimum variance the within-cluster variance will be as small as possible for the most similar clusters. The distance between the clusters was measured with the squared Euclidean distance.

The final step of the analysis was to assess long-term outcomes for every individual phenotype.

The ACQ6, the AQLQ at 12 month-follow-up, the amount of severe exacerbations during the study-period and medication prescription were selected as long-term outcomes. The ACQ6 is a six-question questionnaire aimed to map asthma control, it scores between 0–6 and higher scores represent worse asthma control.^18^ The AQLQ assesses asthma related quality of life, the score has a range from 0 to 7 with higher scores representing a higher quality of life. A severe asthma exacerbation was defined as a course of oral prednisolone prescribed for worsening asthma for three or more days, or an emergency department visit/hospitalisation due to asthma.^18^

Medication prescription was defined as the total usage of ICSs, recalculated to beclomethasone equivalent dosage. Additionally the use of long-acting beta agonists was presented separately. Finally all medication usage was also classified into one ‘medication step’, based on the steps presented in the NAEPP guidelines.^32^

### Data availability

Data analysed in this study are available upon request.
